# Employees and the National Insurance Act

**Published:** 1912-08-10

**Authors:** 


### NATIONAL INSURANCE ACT DEPARTMENT.

### EMPLOYEES AND THE NATIONAL INSURANCE ACT.

### XXIII.—The Insurance of Aliens.

Aliens between the ages of sixteen and seventy who are in employment and receive remuneration at the rate not exceeding £160 per annum must be insured in the same way as British subjects. That is to say, their employers are liable to pay the same contributions as though they were British subjects, but there are special conditions regulating the right to benefits. These conditions are not only of interest to aliens themselves, but also to the general body of insured workers, because by comparison with the ordinary rates and conditions of benefit the special advantages derived by British subjects entering insurance at ages above sixteen are thrown into prominence.

The special provisions as to aliens are set out in Section 45 of the Act, and the rates of benefit described are those contained in a circular numbered A.S. 23 recently issued by the Insurance Commissioners.

Aliens not Affected by the Special Provisions.

The following persons are not treated as aliens but are entitled to insurance on the same conditions and at the same rates as British subjects:—

1. An alien entering insurance under the age of seventeen.

2. An alien who becomes or who has become a naturalised British subject.

3. An alien who, on May 4, 1911, was a member of a society which becomes an approved society, and who at that date had been resident in the United Kingdom for five years or upwards. It is important to note in this connection that the membership of a society is satisfied whether such society is a friendly, dividing or collecting society, or an industrial insurance company.

4. A woman who, having been a British subject before marriage, has married an alien, and whose husband subsequently dies, or whose marriage is dissolved or annulled, or who has for at least two years been separated from or deserted by her husband.

5. A person transferred to an approved society or the Post Office Fund in pursuance of an arrangement with the Government of any foreign State.

The Object of the Exceptions.

These exceptions from the operation of the special provisions are designed to enable as many as possible to be brought into the full benefits of insurance. In the case of persons under the age of seventeen at entry into insurance no initial reserve has to be set up for those who become members of approved societies. No special advantage is, therefore, given to persons entering insurance at the age of sixteen beyond the State grant of two-ninths of the benefits in the case of men and one-quarter in the case of women. For some years to come this State grant is being exactly counterbalanced by a deduction from each weekly contribution of one penny and five-ninths (or in the case of women one penny halfpenny) for the purpose of discharging the liabilities of the Insurance Commissioners to approved societies in respect of the reserve values set up for those entering insurance above the age of seventeen. Thus as at the age of sixteen the value of the contributions is exactly the same, on the basis of the actuarial estimates, as the value of the benefits, it would have been doing an injustice to aliens entering insurance at that age to have treated them differently in any respect from British subjects.

An alien who becomes a naturalised British subject obtains the full rights and privileges of his adopted nation, and as a matter of course he must be treated exactly on the same footing as a person who is a British subject from birth.

The third exception above described is in the nature of a special concession, and was incorporated into the Act during its passage through Parliament in order to protect the interests of those aliens who, though they have not become naturalised, have adopted the United Kingdom as their permanent domicile and had made provision for sickness or death without any special inducement from State aid, prior to the introduction of the Bill into Parliament, through a society that becomes approved.

The exceptions numbered (4) and (5) follow as a matter of course, but it should be noted that as on marriage a woman adopts the nationality of her husband, it may happen that a woman who, prior to her marriage, was a British subject and insured at ordinary rates as an employed contributor, will, on her marriage with an alien, while still remaining in employment, suffer reduction of benefits.

The Special Conditions Explained.

Deposit Contributors.

Having described the classes of aliens expressly exempted from the operation of the special provisions of the Act, we must set out the nature of altered conditions of insurance as they affect aliens generally. The principal condition is that no part of the benefits to which such persons may become entitled is to be paid out of moneys provided by Parliament. This applies equally to those who join approved societies and to those who become deposit contributors. In the case of deposit contributors to the Post Office Fund the alteration is effected by the reduction of the rates of sickness, disablement, and maternity benefits, in the case of men to seven-ninths, or in the case of women to three-quarters, of the rate to which they would otherwise have been entitled under the Act. It is further laid down that no part of the sums required for medical and sanatorium benefits nor of the charges for the administration of benefits shall be paid out of moneys provided by the State.

### Members of Approved Societies.

When an alien who is subject to the special provisions joins an approved society the contributions paid on his behalf as evidenced by the stamps affixed to his insurance cards will be credited to his society in the books of the Insurance Commissioners, and by way of benefit he will be entitled to medical and sanatorium benefits as though he were a British subject. In order that these benefits may be provided in full it will be necessary for the approved society to pay the same sum to the Insurance Committee responsible for the administration of the benefits as for a British subject, thus the sum remaining for the provision of sickness and disablement allowances and for maternity benefit is to the same extent depleted.

The rates and conditions of these latter benefits are such as may be determined by the society, but having regard to the fact that the solvency of approved societies is one of the chief considerations of the Insurance Commissioners it is required that such rates as may be offered shall have the sanction of the Commissioners. In order to guide societies in this respect the Commissioners have themselves drawn up tables of suggested rates which are incorporated in the Circular A.S. 23 already mentioned. These rates are not binding on all societies, but if any others are adopted in their place they must first be submitted for the approval of the Commissioners, together with a certificate setting forth the basis on which they are founded in order that their actuarial soundness may be investigated.

### The Rates of Benefit for Men.

In the following schedule we give an extract from the table of rates suggested by the Commissioners for males as applicable in Great Britain:—

Rates of Benefit for Aliens (Males).

| Age at Entry into Society. | Sickness Benefit. Rate per Week. | | Disablement Benefit. Rate per Week. | | Maternity Benefit. | | |
|---|---|---|---|---|---|---|---|
| | s. | d. | s. | d. | £ | s. | d. |
| 17-19 ... | 9 | 6 | 4 | 9 | 1 | 8 | 6 |
| 19-21 ... | 9 | 0 | 4 | 6 | 1 | 7 | 0 |
| 25-30 ... | 7 | 6 | 3 | 9 | 1 | 2 | 6 |
| 35-40 ... | 6 | 0 | 3 | 0 | 1 | 0 | 0 |
| 45-50 ... | 4 | 6 | 2 | 3 | 1 | 0 | 0 |
| 55-60 ... | 2 | 6 | 1 | 3 | 1 | 0 | 0 |

Even a cursory examination of this table shows at once the immense value of the State grant in aid of the benefits applicable to British subjects, when combined with the arrangement for setting up the initial reserves to enable those above sixteen years of age to be taken into insurance at the rate of contribution applicable to that age.

### Benefits for Women.

The corresponding table for females is divided into two main sections according as the woman is at the time of entry (1) a spinster or a widow or (2) a married woman. The necessity for the double classification arises through the provision of maternity benefit. A modified example will suffice to show the difference in the rates. Whereas in the case of alien spinsters and widows entering insurance at ages above twenty-one and under twenty-five the rates of sickness and disablement benefits per week are 6s. 6d. and 4s. 3d. respectively and a maternity benefit of £1 5s. 6d., where payable, the corresponding benefits in the event of the woman being married at the time of entry are sickness and disablement allowances of 4s. 6d. and 3s. respectively, together with a maternity benefit, where this is payable, of 18s. These benefits compare with sickness and disablement allowances of 7s. 6d. and 5s. a week respectively, and a maternity benefit of £1 10s. for all insured women who are British subjects. Thus we, again, have furnished a concrete and instructive illustration of the advantages conferred under the Act. The benefits suggested by the Commissioners become payable at the times and under the conditions expressed in the rules for British subjects, and the sickness and disablement allowances are subject to reduction in the case of unmarried minors, having no dependents, in the same manner, as also when the contributions are in arrear.

It is likely that a small amount of State aid may be given towards the insurance of aliens, for it will be remembered that when low wages are paid and board and lodging are not provided the weekly contributions are on a lower scale than the normal, and in certain cases the sum of 1d. a week is provided by money derived from Parliament. It is notorious that aliens are content to work for low rates of remuneration, and, although it was apparently the intention to provide State aid for British subjects only, the wording of the section seems to indicate that it is only in regard to the proportion of the benefits paid, and not as regards contributions, that Parliamentary funds will not be available.

### INSURANCE OF PRIVATE NURSES.

In a recent issue we advised one of our correspondents with a private nursing practice to write to the Insurance Commissioners for an authoritative decision as to whether she had to be insured. She has very kindly furnished us with a copy of the reply she received in the following terms :—

"The National Health Insurance Commission (England) are advised that, generally speaking, nurses working on their own account will be required to be insured, and the persons by whom they are engaged will, as the employers, be liable for the payment of the contribution. The contributions due from the nurse will be recoverable by deduction from her remuneration; during unemployment it will be open to the nurse to pay the whole contribution herself to avoid reduction of benefits owing to arrears. The only case, generally, in which a nurse will not have to be insured (subject to the ordinary provisions of the Act as to casual employment and employment at a rate of remuneration exceeding in value £160 a year) is the case where she receives patients into her own house for treatment."

This is a definite pronouncement, and, being in general terms, would seem to apply not only to the case in point, but to all nurses.

NOTE.—Questions and Correspondence on all doubtful points are invited, and should be addressed to the Editor, The Hospital, 28 Southampton Street, Strand, W.C., and marked "Insurance."

